# Promoting deceased organ and tissue donation registration in family physician waiting rooms (RegisterNow-1 trial): study protocol for a pragmatic, stepped-wedge, cluster randomized controlled registry

**DOI:** 10.1186/s13063-017-2333-5

**Published:** 2017-12-21

**Authors:** Alvin H. Li, Amit X. Garg, Versha Prakash, Jeremy M. Grimshaw, Monica Taljaard, Joanna Mitchell, Danny Matti, Stefanie Linklater, Kyla L. Naylor, Stephanie Dixon, Cathy Faulds, Rachel Bevan, Leah Getchell, Greg Knoll, S. Joseph Kim, Jessica Sontrop, Lise M. Bjerre, Allison Tong, Justin Presseau

**Affiliations:** 1 Clinical Epidemiology Program, Ottawa Hospital Research Institute, Ottawa, ON Canada; 2 Lawson Health Research Institute, London, ON Canada; 3 Institute for Clinical Evaluative Sciences (ICES), Toronto, ON Canada; 4 Department of Epidemiology and Biostatistics, Western University, London, ON Canada; 5 Division of Nephrology, Western University, London, ON Canada; 6 Trillium Gift of Life Network, Toronto, ON Canada; 7 Department of Medicine, University of Ottawa, Ottawa, ON Canada; 8 School of Epidemiology and Public Health, University of Ottawa, Ottawa, ON Canada; 9 Institute of Health Policy, Management, and Evaluation, University of Toronto, Toronto, ON Canada; 10 Department of Family Medicine, Western University, London, ON Canada; 11 University Health Network, University of Toronto, Toronto, ON Canada; 12 School of Psychology, University of Ottawa, Ottawa, ON Canada; 13 Department of Family Medicine, University of Ottawa, Ottawa, ON Canada; 14 Sydney School of Public Health, The University of Sydney, Sydney, NSW Australia

**Keywords:** Organ and tissue donation, Behavior change, Stepped-wedge trial, Cluster randomized trial, Organ and tissue donor registration, Protocol

## Abstract

**Background:**

There is a worldwide shortage of organs available for transplant, leading to preventable mortality associated with end-stage organ disease. While most citizens in many countries with an intent-to-donate “opt-in” system support organ donation, registration rates remain low. In Canada, most Canadians support organ donation but less than 25% in most provinces have registered their desire to donate their organs when they die. The family physician office is a promising yet underused setting in which to promote organ donor registration and address known barriers and enablers to registering for deceased organ and tissue donation. We developed a protocol to evaluate an intervention to promote registration for organ and tissue donation in family physician waiting rooms.

**Methods/design:**

This protocol describes a planned, stepped-wedge, cluster randomized registry trial in six family physician offices in Ontario, Canada to evaluate the effectiveness of reception staff providing patients with a pamphlet that addresses barriers and enablers to registration including a description of how to register for organ donation. An Internet-enabled tablet will also be provided in waiting rooms so that interested patients can register while waiting for their appointments. Family physicians and reception staff will be provided with training and/or materials to support any conversations about organ donation with their patients. Following a 2-week control period, the six offices will cross sequentially into the intervention arm in randomized sequence at 2-week intervals until all offices deliver the intervention. The primary outcome will be the proportion of patients visiting the office who are registered organ donors 7 days following their office visit. We will evaluate this outcome using routinely collected registry data from provincial administrative databases. A post-trial qualitative evaluation process will assess the experiences of reception staff and family physicians with the intervention and the stepped-wedge trial design.

**Discussion:**

Promoting registration for organ donation in family physician offices is a potentially useful strategy for increasing registration for organ donation. Increased registration may ultimately help to increase the number of organs available for transplant. The results of this trial will provide important preliminary data on the effectiveness of using family physician offices to promote registration for organ donation.

**Trial registration:**

ClinicalTrials.gov, ID: NCT03213171. Registered on 11 July 2017.

**Electronic supplementary material:**

The online version of this article (doi:10.1186/s13063-017-2333-5) contains supplementary material, which is available to authorized users.

## Background

There is a worldwide shortage of organs available for transplant. In 2013, approximately 4400 Canadians were on a waiting list for an organ transplant and 246 died waiting [1]. Many of these deaths could be prevented if more organs were available for transplant [1]. Many countries, including Canada, use an “opt-in” system, where a person can record in an electronic registry their desire to become an organ donor upon their death. The decision to donate organs ultimately falls to the decedent’s next of kin; however, knowing whether their loved one registered for organ and tissue donation can help relatives make this difficult and time-sensitive decision during the grieving period [2]. The availability of an opt-in organ donation register allows an organ donation coordinator to determine if the deceased was a registered donor before approaching their family. The coordinator can then share this information with the family member and ask if they would like to reaffirm that choice. In the province of Ontario in Canada, an estimated 90% of families consent to donation when their deceased loved one is a registered organ donor, compared to 50% who consent when the deceased had not registered [3]. Up to 90% of Canadians support organ donation; however, the proportion of Canadians actually registered is substantially lower and varies by provinces and territories [4].

In Ontario, individuals typically register for organ donation where they obtain or renew their driver’s licence or health card [5]. While prompting individuals to register at these locations is an important part of an overall strategy, given low registration rates, more opportunities to prompt members of the public to consider donor registration are needed [6]. For many reasons, the family physician office is a promising, yet underused and under-evaluated, additional setting with potential for promoting and prompting organ donor registration. Indeed, many individuals report that their family physician is a trusted source of information for organ donation and view family physician offices as an appropriate setting to obtain information about organ and tissue donation [7]. Patients are already thinking about health issues when visiting their family physician and more so relative to other settings in which organ donation might be promoted (e.g., departments of motor vehicles or large gatherings such as sporting events). Family physicians believe that discussing organ donation with their patients is within their scope of practice, although they may they lack the time to do so themselves [8]. There is also an opportunity for patients to take a few minutes to register for organ donation while waiting to see their family physician.

Several studies examining the effect of interventions to increase organ donor registration conducted in family physician settings report promising findings [6, 9–11]. Salim et al. compared the effects of a staffed kiosk containing organ donation educational material and Donor Registry Forms compared to an unstaffed kiosk in the waiting room [9]. During the unstaffed 6-week period, they found that only two patients registered over a total of 59,181 patient encounters at four clinics. During the staffed week, 102 patients registered over 9805 patient encounters. These findings emphasize that having an interpersonal component may be important in promoting organ donation registration. Bidigare et al. conducted a trial in one family practice, randomizing 300 patients to receive an information brochure or a brochure combined with a brief verbal discussion with the family physician encouraging completion of organ donor cards [10]. Thirty-three percent of patients in both arms had already committed to organ donation via driver licence stickers. Of those remaining, 40% in both arms intended to sign a donor card after the intervention, with both approaches being equally effective. Such findings suggest that while verbal discussions may be beneficial, they do not alter intentions to register for organ donation relative to a delivery method involving only a brochure. Thornton et al. conduced a randomized trial in 18 primary care offices involving 915 patients who have not yet registered for organ donation [6]. The intervention group watched a 5-min organ donation video in the waiting room and selected a question to ask their physician about organ donation (*n* = 456). The control group was consented and enrolled into the study but received usual care (*n* = 459). Intervention patients were more likely to register for organ donation (22% new registrations) compared to 15% new registrations in the control which simply involved enrolling in the study. Across all the above-mentioned studies, the emphasis has been on comparing methods of delivering intervention rather than the intervention content itself. The descriptions lacked details on the actual content of the intervention, i.e., behavior-change techniques (BCTs) delivered to address barriers and enablers to registration. Such details remain under-reported and under-specified in this academic literature leading to uncertainties in whether the reported effects are a function of any video-, brochure-, or verbal-delivery method, and/or the techniques delivered within these modalities. Further research with more detailed description is needed to facilitate the generalizability, replication and optimization of future interventions promoting organ donor registration.

### Aims and objectives

Our aim is to develop and evaluate an intervention designed to address identified key barriers and enablers to registering for organ donation. This protocol describes the development of our intervention and the design and methods that we will use to evaluate the effectiveness of our intervention in increasing organ donation registration.

Our primary objective is to evaluate whether a behavior-change, theory-based intervention developed in partnership with citizens, family physicians, and a provincial organ procurement agency delivered to six family physician offices can increase registration for deceased organ donation. The two main components of the intervention involve (1) reception staff providing unregistered patients with a pamphlet addressing barriers and enablers to organ donation registration and (2) an opportunity for immediate registration using an Internet-enabled tablet in the waiting room. Our secondary objective is to conduct a process evaluation to assess the experience of delivering the intervention from the perspective of family practice staff.

### Trial design

This is a protocol for a stepped-wedge, registry-based, cluster randomized trial conducted under real-world settings. The stepped-wedge design has the advantage of robustly evaluating the effectiveness of the intervention while also allowing all participating sites to receive the intervention. A stepped-wedge design was adopted because we suspected it to be unlikely that family physicians would agree to be randomized unless they were guaranteed, at some stage during the trial, to receive the intervention. Family physician volunteers recruited to the trial are likely to have personal connections and interests in organ donation. In a parallel arm design, being allocated to the control arm could have resulted in disappointment and may have prompted physicians to drop out of the study or adopt other interventions to promote organ donation.

The registry-based design uses routinely collected data to ascertain our outcomes. In Ontario, Canada, we have comprehensive, routinely collected healthcare administrative databases that are linked via encoded identifiers to facilitate health research (e.g., Ontario’s organ donor registry and family physician billing records). It is a cluster trial in that the unit of randomization is family physician offices (where each office may be staffed by several family physicians). Cluster randomization was used because the nature of the intervention precludes individual randomization. The order in which six family physician offices (the clusters) will receive the intervention will be randomly allocated. We used the Standard Protocol Items Recommendations for Interventional Trials (SPIRIT) Checklist to guide the reporting of our protocol (Additional file 1: Table S1) [12], the Template for Intervention Description and Replication (TIDieR) to guide the reporting of components of our intervention [13] and the Behavior Change Techniques Taxonomy version 1 (BCTTv1) to describe which BCTs we are employing in our intervention [14].

## Methods

### Participants, interventions and outcomes

#### Study setting

We will conduct the trial in six family physician offices located in Ontario, Canada. Key components of the trial are described in Table 1. As of 2008, Ontario’s organ and tissue donor registry became affirmative only (i.e., recording only “yes” responses). Citizens of 16 years of age and older can register online or can mail in a Donor Registration Form. It is also provincially mandated that individuals be asked about organ and tissue donor registration with all health-card related transactions, driver’s licence renewals and photo ID applications at ServiceOntario centers. Those who choose to register can also select to donate for research purposes and exclude certain organs or tissues from donation. While citizens can also withdraw their registration whenever they like, less than 0.1% of the registered population withdraws its registration each year.

**Table 1:** Key characteristics of the trial

Trial characteristics	Definition
Cluster (unit of randomization)	Family physician office (a total of 6)
Number of sequences (steps)	6 (one office per sequence)
Duration of trial	14 weeks
Number of measurement periods	7 (length of each period is 2 weeks)
Individuals	Patients eligible to register for deceased organ donation and visiting a family physician at any time during the study
Timing of start of exposure	Individuals are exposed in a continuous and gradual process as they present to their family physician offices
Duration of exposure	All individuals are exposed for a short period during their visit to the physician office
Measurement	Repeated measurements are taken from mostly different individuals in each period; it is possible that a very small proportion of individuals will have repeat visits to their family doctors but because no identifying information will be collected, such individuals will be included in the analysis as independent individuals
Total number of clinics (clusters)	6

#### Eligibility criteria for family physician offices (clusters) and family physicians

We will enroll family physician offices that see a minimum of 100 patients per week (see sample size calculation for justification). If it arises that multiple physician offices share the same waiting room, these will be considered as a single site given that all patients within the waiting room will be exposed to the intervention. We will include family physicians from the enrolled site who are willing to provide their Canadian Physician Surgeons Ontario number (a personal identifier) to allow linkage between the billing date for fee for service payments for patient visits and the date of donor registration. We will exclude family physicians if they primarily work at multiple sites because our administrative databases only allow linkage of patients to physicians but not to the location of patient visits.

#### Eligibility criteria for patients

For our outcome analysis using routinely collected registry data, we will include any patients aged 16 years and older with a valid health card (all of whom are considered eligible to register for deceased organ in Ontario). These patients will have at least one outpatient visit during the period of interest with a family physician participating in this trial.

#### Intervention development and description

To inform the development of this intervention, we identified theory-based barriers and enablers to organ donation registration. We conducted semi-structured interviews with 20 patients from one family practice (nine of which had not registered for organ donation). The interview guide was based on the Theoretical Domains Framework [15]. Findings showed that patients were largely aware and motivated to register for organ donation. However, many patients were unclear on how to register and whether they were eligible and did not realize that their donor status is on the back of their health card. In Ontario, Canada, the donor registration status is found on the health card rather than the driver’s licence. It reflects registration status at the time that the card was issued, where cards need to be renewed every 5 years. Many patients also assumed their health status made them a poor candidate. That said, many were clear on the benefits of registration to themselves and others. Importantly, a key barrier involved a lack of priority and not getting around to it. We therefore leveraged these findings to design an intervention to provide an opportunity to register, to address barriers and enablers and to prompt them to consider registration in the waiting room of a family physician office as a novel and suitable setting for considering this health decision (see Additional file 2: Table S2).

We then identified possible methods of delivery that can be feasibly implemented within a family physician waiting room. Considering the time constraints and competing demands faced by many family physicians in their office, we identified practice reception staff as a resource for providing components of the intervention. Receptionists are the first point of contact that patients have with the practice, which inherently involves requesting the health card at the time a patient presents to the counter upon arrival. As donor status is listed on the health card, it can be easily verified. Reception staff are ideally positioned for providing physical materials (pamphlets) and prompting the use of tablets or cellphones in the waiting room. We linked possible BCTs to identified barriers and enablers from the interviews. We used an iterative process to develop and operationalize BCTs and methods of delivery with stakeholders (citizen panel, researchers, family physicians and provincial organ procurement organization). Intervention materials including the training protocols will be uploaded to our website and included with the trial results publication.

#### Detailed description of the intervention

The intervention involves three key components: (1) case finding by reception staff to identify patients who have not yet registered for organ donation by checking the back of their health card, (2) reception staff providing pamphlets designed to deliver BCTs designed to address previously identified barriers and enablers and (3) providing an Internet-enabled tablet in the waiting rooms to enable immediate and secure online registration while waiting for their appointment [16, 17]. The theoretical basis of the intervention is consistent with Social Cognitive Theory and triadic reciprocal determinism between the person (and their cognitions), their behavior and their environment [18, 19], and a basis in a dual process model of behavior change emphasizing a role for reflective as well as impulsive, automatic influences on behavior through changes in the physical and social environment and prompts/cues to behavior [20, 21]. Table 2 describes the BCTs to be delivered within each intervention component, linked to the specific barriers and enablers targeted by each technique as described by the Theoretical Domains Framework, and outlined in more detail below [14, 15, 22].

**Table 2:** RegisterNow-1 intervention description

Component 1: case finding		Component 2: pamphlet		Component 3: immediate opportunity to register	
Who delivered?: Reception staff		How delivered?: Paper pamphlet provided by: reception staff		How delivered?: Tablet	
BCT	Domain	BCT	Domain	BCT	Domain
Instruction on how to perform the behavior	Knowledge (procedural)	Instruction on how to perform the behavior	Knowledge (procedural); skills; beliefs about capabilities	Adding objects to the environment	Beliefs about capabilities; environmental context and resources; memory, attention and decision processes; behavioral regulation
Social support (practical)	Knowledge (procedural)	Information about others’ approval	Social influences; goals; emotion; beliefs about consequences	Prompts/cues	Memory, attention and decision processes
Prompts/cues	Memory, attention and decision processes	Credible source	Social influence; emotion; beliefs about consequences		
Information about others’ approval	Social influences	Social comparison	Social influences		
		Prompts/cues	Memory, attention and decision processes		
		Verbal persuasion of capability	Beliefs about capabilities; Knowledge		
		Vicarious consequences	Beliefs about capabilities; beliefs about consequences; emotion		
		Information about social and environmental consequences	Beliefs about consequences; social influences; social/professional role and identity		
		Salience of consequences	Beliefs about consequences; social influences		
		Information about emotional consequences	Beliefs about consequences; emotion		

*BCT* behaviour-change technique, *Domain* specific barrier/enabler targeted by the BCT, based on domains described by the Theoretical Domains Framework

##### Component 1 – Reception staff case finding

First, reception staff will verify the organ donor registration status of patients upon their arrival at the clinic on the provincial health card that patients must provide to receive healthcare services from their family physician. As reception staff already request a patient’s health card during their visit, this step is designed to fit within existing work routines rather than increasing any workload. Reception staff will provide instruction on how to perform the behavior, social support (practical) and prompts/cues to address citizens’ procedural knowledge about how to check whether they are registered and how to register, and to prompt them to do so if they wish. They will provide a pamphlet and indicate that they can register while they wait by using the tablet in the waiting room or their cellphone.

##### Component 2 – Paper pamphlet

A paper pamphlet provided by reception staff to patients whose health card does not indicate that they are registered donors will aim to directly address barriers and enablers to registration. The pamphlet will also be available in each waiting room for any patient to take if they wish. The pamphlet will provide visual and written instruction on how to check donor status and how to register securely online, will provide information about others’ approval using credible sources (using photos, signatures and stories from family physicians and local patients), will facilitate social comparison with other local patients, will persuade them of their capability to register by indicating how easy it is and how little time it takes, and then providing information of vicarious and salient consequences of registering (impact on organ transplant recipients), information on social and environmental consequences (impact on the wider community), and information on emotional consequences (how they may feel once registered).

##### Component 3 – Providing immediate opportunity to register

A secure Internet-enabled tablet (i.e., an iPad) will be added in each waiting room to give patients the immediate opportunity to register for organ donation online via a secure provincial website. Adding the tablet also aims to address beliefs about capability to register and acts as a prompt/cue to register, focusing their attention and prioritizing registration while they wait. Table 3 provides a further overview of intervention as per TiDIER criteria, and online additional materials provides detailed mapping of BCTs to theoretical domains informed by interview results). All materials will be in English only for this intervention. The location of the materials will be tailored according to the family physician office’s preferences.

**Table 3:** Overview of the RegisterNow-1 intervention, as per Template for Intervention Description and Replication (TiDIER) criteria

TiDIER criteria	Description of intervention and quality control procedures
Brief name	RegisterNow-1 intervention
Why?	Patients are often unsure if they registered for organ donation. Many patients support organ donation but have not prioritized it and may not get around to it. Providing immediate opportunity to register for organ donation would address this barrier and leverage those already motivated to register
What materials?	Pamphlets designed to address previously identified barriers and enablers to organ donation registration, Internet-enabled tablet, and training material for office staff
What procedures?	Reception staff will check health cards for donor status, use a standard script to provide a pamphlet to patients and suggest to those that have not yet registered for organ donation that they can do so using the tablet in the waiting room. The pamphlet will describe ways the patient can register for organ donation (e.g., use the Internet-enabled tablet in the waiting room). Behavior Change Techniques (BCTs) are described in Table 2
Who provided?	Reception staff
How?	Face to face, paper and electronic
Where?	Family physician office waiting room
When and how much?	The intervention will be available between 2 and 14 weeks in duration (depending on the randomly allocated start date). For a given patient, they are likely only to be exposed to the intervention once unless they have a repeat visit during the period in which their practice is delivering the intervention
Tailoring	Reception staff can adapt the script to their practice. The content of the pamphlet can be minimally tailored according to the family physician office to include the name of the practice, the name of the physicians at the practice, a photo of the physicians and their signature. The location of the Internet-enabled tablet and additional pamphlets within the waiting room can be tailored by the practice staff

### Enhancing the fidelity of delivery and adherence to the intervention

We will conduct a setup meeting within each family practice, involving family physicians and reception staff. Nurses and nurse practitioners may also be involved depending on the site. This meeting will involve describing the intervention, roles and responsibilities, presenting the materials, clarifying the start date and duration of the intervention and trial design, and tailoring the location of the tablet and the patient-focused pamphlets using practice-specific content (photos, logos, signatures).

Following the setup meeting, a research assistant and/or researcher will conduct a 1-h in-person training session with reception staff in each office, to clarify how the intervention is delivered, the start date, the duration of delivery, and provide instructions to reception staff on how to check all patients’ health cards for their donor registration status at the time of the patient visit, what to say to patients, provide an overview of the content of the pamphlets that they are asked to provide, and an overview of how to setup and use the tablet. These will also be provided in writing for any reception staff not able to attend the session. The in-person training session will occur within 4 weeks prior to the intervention start time in each office. Staff training will be based on the principles of Social Cognitive Theory, focusing on practicing and rehearsing to develop a new routine and self-efficacy to deliver the intervention and providing information on health and social consequences to support the need for the intervention and motivation to deliver it [23]. Staff will also receive paper-based descriptions of suggestions for how to address questions that may arise from patients. For example, some patients may ask for more details regarding the rules around family vetoing deceased organ donation. This booklet will also encourage physicians to talk to their patients about organ donation if appropriate.

On the first day of crossing into the intervention condition, the research assistant will bring all materials to the practice and set up the tablet. A research assistant will contact a representative from each family physician clinic by phone or email the week prior to beginning the intervention period to confirm readiness. Offices will then be contacted again every week until the end of the trial to promote ongoing fidelity of delivery, remind them (prompts/cues) and resolve emerging issues (problem solving). Problems that may emerge may include technical issues such as the tablet not functioning or running out of pamphlets. We will provide each member of family physician office staff who participated in the trial with an honorarium of CAD$50 upon completion of the trial.

### Control condition

The nature of the stepped-wedge design is such that practices will all begin in the control condition and then sequentially cross to the intervention condition for the remainder of the study period. We will ask family physicians and reception staff to continue with their usual practice while in the control condition until the date that they have been randomized to cross to the intervention condition. Intervention materials and training (e.g., pamphlets, tablet) will not be provided until their site crosses into the intervention arm.

### Outcomes

The primary outcome is the proportion of all patients aged 16 years and older who visited the office during each 2-week interval who are registered for deceased organ donation at 7 days following the family physician visit. We chose 7 days to ensure that patients have the opportunity, should they wish, to discuss their decision to register with their family or friends. However, we expect and have designed the intervention so that most of the registrations take place immediately (same day) during their family physician clinic visit; this is consistent with the observed barriers to registration. We designated prevalence of donor registration (rather than incidence of new registrations) as the primary outcome for the trial as it was important to measure the net effect of our cluster-level intervention on the overall prevalence of donor registration among all patients with visits to their family physician. Secondary outcomes are the proportions of unregistered patients aged 16 years and older who newly register for deceased organ donation within 7 days of visiting the family physician clinic, as well as at 1 day, 14 days and 30 days post visit.

### Study timeline

**Fig. 1:**
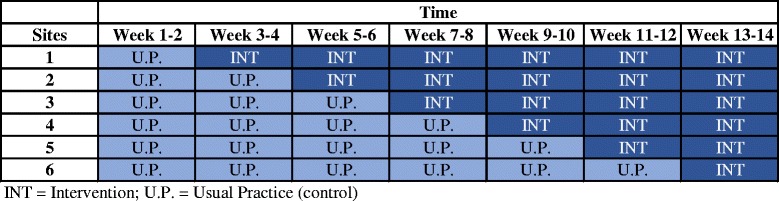
Stepped-wedge design

We will recruit all six family physician offices prior to the start of the intervention (see Fig. 1). Given that there is a delay for the records to enter the administrative databases, we will only be able to assess baseline information and outcomes at the end of our trial.

### Sample size

We would consider an absolute increase of 10% or more in the proportion of patients who are registered organ donors at 7 days post encounter to be both clinically important and realistic to achieve. Our sample size of six clusters (10,500 patients in total) achieves 80% power to detect a 10% absolute difference assuming a control proportion of 0.5 using a two-sided test at the 5% level of significance [24]. Our calculation assumes an intracluster correlation coefficient of 0.06, as calculated from our previous work [Institute for Clinical Evaluative Sciences KDT. Feasibility of organ donor registration in family physician offices across Greater Toronto Area. Internal report: 2015; unpublished.], an average of 250 patient encounters per site in each 2-week interval, and a cluster autocorrelation coefficient of 0.8 to allow for a 20% decay in the strength of the correlation in repeated measures over time [24]. The percentage of registered donors in the control condition is conservatively assumed to be 50% to allow for a higher prevalence of registered donors in our participating offices than the provincial average. No adjustment is made for cluster attrition as the risk of attrition is extremely low, and all outcomes will be assessed from routinely collected sources, regardless of any dropout. Given some uncertainty around parameter estimates required for the stepped-wedge sample size calculation, sensitivity of our detectable effect size to a range of alternative assumptions is presented in Table 4. The results show that across a range of control arm proportions (from 0.4 to 0.5), average cluster sizes (from 100 to 400) and cluster autocorrelation coefficients (from 0.8 to 0.95), our sample size of six practices will achieve 80% power to detect absolute increases of between 5 and 11%.

**Table 4:** Sensitivity analysis

Control proportion	Intervention proportion	Within-period ICC	Cluster autocorre-lation	Average cluster size per period	Absolute increase in proportion
0.5	0.61	0.06	0.8	100	0.110
	0.6	0.06	0.8	250	0.100
	0.59	0.06	0.8	400	0.090
	0.59	0.06	0.95	100	0.090
	0.565	0.06	0.95	250	0.065
	0.56	0.06	0.95	400	0.060
0.4	0.51	0.06	0.8	100	0.110
	0.5	0.06	0.8	250	0.100
	0.49	0.06	0.8	400	0.090
	0.49	0.06	0.95	100	0.090
	0.465	0.06	0.95	250	0.065
	0.46	0.06	0.95	400	0.060
0.45	0.56	0.06	0.8	100	0.110
	0.55	0.06	0.8	250	0.100
	0.545	0.06	0.8	400	0.095
	0.54	0.06	0.95	100	0.090
	0.515	0.06	0.95	250	0.065
	0.51	0.06	0.95	400	0.060

Sensitivity analysis showing detectable difference with a sample size of six practices, expressed as an absolute increase in proportions, with 80% power using a two-sided test at the 5% level of significance

### Recruitment

We will recruit a convenience sample of practices from within our network of family physician office contacts within the London, Ontario (41% donor registration rate) and Stratford, Ontario communities (46% donor registration rate). A collaborating family physician will send an introductory email to potential family physician contacts, inviting them and their practice to consider participating. We will then arrange an in-person meeting with family physicians from interested sites to introduce our study and obtain written agreement from family physicians and offices agreeing to participate that meet our eligibility criteria.

### Sequence generation (i.e., randomization)

**Fig. 2:**
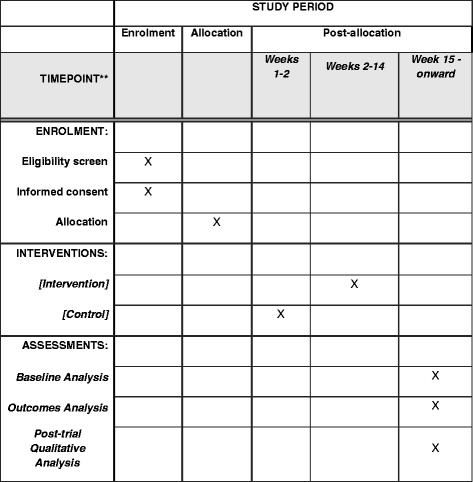
Trial schedule of enrollment, intervention and assessment (as recommended by SPIRIT; Figure displaying schedule of enrollment and interventions)

Each of the six participating clinics (clusters) will be randomly allocated to six different starting dates for the intervention (steps). A statistician blinded to cluster identity and not involved in the intervention delivery will generate the allocation sequence using computer-generated random numbers. The timing of the transition from control to intervention will not be communicated to the offices and the research coordinator until 2–4 weeks prior to transitioning (to balance minimizing risk of bias during control periods with the need for planning, training and setup for implementing the intervention) (see Fig. 2 for the SPIRIT Figure). The research coordinator will communicate intervention starting times with the participating clinics.

### Blinding

Family physicians and their staff will be aware of the intervention they provide during the intervention periods (i.e., they are not blinded). However, the timing of allocation to intervention will be concealed from investigators, participating physicians and their patients. In addition, the biostatistician performing the analysis will be blinded to the identity of the sites. In both the control and intervention condition, family physician offices will have a poster in the waiting room that the site is currently participating in a research study and feature a link for more information, but patients will not be explicitly told which condition they are in.

### Data collection methods/data management

We will ascertain baseline and outcome information from healthcare administrative databases housed at the Institute for Clinical Evaluative Sciences (ICES). Under section 45 of Ontario’s Personal Health Information Privacy Act, ICES is considered a prescribed entity. Health information custodians, such as physicians, can disclose personal health information to ICES without patient consent for the purpose of research. These databases can be linked via encoded identifiers. Specifically, we will use our list of recruited family physicians to link to the Ontario Health Insurance Plan (OHIP) billing datasets to identify the patients who visited our recruited family physicians and the dates at which they visited. We will then use the Registered Persons Database to obtain patient demographic information and their donor registration information (including date of registration).

### Additional analyses

We will also conduct several pre-specified subgroup analyses to examine the differential effect of our intervention by age (younger (≤40 years) vs. older (>40 years)) and sex (male vs. female). We hypothesize that our study may be more effective among older individuals who tend to have more concerns about their eligibility to register for organ donation (e.g., too old to register). We will explore differential intervention effects by sex but hypothesize that there are no differences between male and female.

### Statistical methods

We will describe site and patient demographic characteristics across all control and intervention periods using descriptive statistics. All analyses will be conducted according to the intention-to-treat principle. We will analyze the primary and secondary outcomes at the individual patient-level using mixed-effects logistic regression accounting for the stepped-wedge design as described by Hooper et al. [25]. The model will include intervention status and time as fixed effects and will include a random intercept and slope for time defined at the level of the family physician office. The inclusion of these two random effects accounts for within-period and between-period intracluster correlations. To correct the potential inflation of the type I error rate due to small number of clusters, we will use the Kenward and Roger method [26]. To express the estimated effect of the intervention as an absolute difference, we will fit the regression model with the binomial distribution and an identity link function; in case of non-convergence, the log or logit link function will be used and results will be reported as either rate ratios or odds ratios together with 95% confidence intervals. With only six offices in our trial, there may be differences in the characteristics of patients visiting the offices during the periods of interest that may confound the intervention effect. We will adjust for three patient-level covariates: age, sex and neighborhood income quintile. Our previous studies showed that younger age, female sex and higher income quintile were associated with organ donor registration [27]. Subgroup analyses will be conducted either as stratified analyses or by including interactions between each subgroup variable, time and the intervention indicator. We will conduct all analyses using Statistical Analysis Software (SAS) version 9.4 and statistical significance will be assessed at the 5% level.

### Timeline

We planned to begin our trial in September 2017. We will then compile, link and analyze the data from our data sources. We will aim to complete our primary outcome analysis by June 2018.

### Data monitoring, harms and auditing

We do not have a Data Safety and Monitoring Board because our trial has a short duration and poses minimal risk. We do not plan to conduct any interim analyses for our primary outcome. The research assistant will track any unintended effects of the trial intervention and conduct based on the weekly calls with the participating sites. Unintended effects may include patients feeling uncomfortable receiving a pamphlet on organ donation. We will not conduct any audits of trial conduct (i.e., review of core trial processes and documents).

### Qualitative post-trial process evaluation

While the trial is designed to evaluate whether the intervention was effective in increasing registration for organ donation, the trial itself is not designed to explain the experiences of the primary healthcare staff involved in delivering the intervention and being involved in the stepped-wedge trial. Process evaluations alongside cluster randomized trials are increasingly being recommended [28] to better understand any underlying factors that may explain the findings of the trial [29, 30]. Consistent with these recommendations and following completion of the trial, we will conduct a qualitative post-trial process evaluation to assess family physicians, nurses and receptionists’ views and experiences of the trial.

### Design, practice staff and sample size

After completion of the trial, we will conduct qualitative interviews with healthcare and administrative staff in trial practices. We will conduct one-to-one, semi-structured interviews with at least one physician, one nurse and one receptionist from each participating family physician office. The sample size is necessarily opportunistic based on the number of physicians, nurses and receptionists in each office but given past experience conducting similar interviews, will likely not exceed 20 interviews; we will use the “10 + 3” rule to determine data saturation, conducting at least 13 interviews, continuing to interview until three consecutive interviews are conducted where no new themes emerge (our past research in similar contexts has typically shown that saturation is achieved within 15 to 20 interviews).

### Recruitment

At the end of the trial period, a member of the research team will contact each family physician office contact from the trial, who will be asked to send an email invitation, including participating information, to all practice staff on behalf of the trial team. Interested participants will be asked to contact the study research assistant directly by email or telephone if interested to set up a time and place to conduct the interview.

### Procedure

Interviews will be conducted at a time and place of convenience to participants, either in person or by phone. Participants will be asked to complete and sign a Consent Sheet. Interviews will be audio-recorded, transcribed verbatim, then anonymized.

### Topics covered

The interview topic guide and coding manual will be framed based on the Theoretical Domains Framework [15, 22]. We will also cover topics including overall experience of the intervention, of the stepped-wedge trial design itself, and adherence to intervention delivery by means of self-report delivery. For reception staff, the interview will focus on their provision of the pamphlet and whether they indicated the registration opportunity (e.g., tablet) to patients. For clinical staff, the interview will focus on experiences with any follow-up discussions with patients concerning organ donation. This includes conversations that took place either in the waiting room or the medical assessment room.

### Planned analyses

Anonymized transcripts will be independently double coded by two researchers using directed content analysis [31] based on the Theoretical Domains Framework following published recommendations [32].

## Ethics

We will follow the Ottawa Statement on the ethical design and conduct of cluster randomized trials [33]. Our intervention will be delivered at the cluster level (family physician offices) and we were approved for an alteration of individual patient consent. The Tri-council Policy Statement on the Ethics for Research Involving Human Participants indicates that an alteration of individual informed consent is permitted when the research involves minimal to no risk to participants [34]. The alteration is unlikely to adversely affect the welfare of participants, and it is impracticable to carry out the research given the cluster-level intervention and research design if the prior consent of participants is required. Specifically, we have asked our Research Ethics Board for a waiver of the requirement to obtain informed consent for study participation from patients. We obtained a waiver of consent for data collection because we are not obtaining any identifiable private information from patients. Information will be obtained using encoded, linked, administrative healthcare databases housed at ICES (see the “Data collection” section). We obtained a waiver of consent for study intervention because it was deemed that our intervention posed minimal to no risk to patients. Patients are routinely offered educational materials on organ donation in other settings such as workplace campaigns, sporting events and when they renew their licence or health card in-person. Family physicians will serve as gatekeepers who provide permission for their clinic to be enrolled in our study. We will also obtain written consent to obtain and link their information to ICES to identify patients who visit their clinic during the course of the study. We will provide a small poster at each office during both intervention and control period informing patients that the office is currently participating in a study on organ donor registration. We will not be able to identify individual patients within the routinely collected data, nor will we collect any identifiable information from patients at the time of their office visits; therefore, patients cannot opt out of the study.

We received ethics approval from the Western University Health Sciences Research Ethics Board to conduct the intervention. We will obtain ethics approval to obtain encoded baseline and outcome data on patients from Sunnybrook Health Sciences Research Ethics Board. Sunnybrook Health Sciences Center provides approval for research projects using the health administrative databases housed at ICES. We will seek ethics approval from the Western University Health Sciences Research Ethics Board for our process evaluation.

### Consent, confidentiality and access to data

Only ICES personnel will have access to the patient-level dataset. Data are also encoded according to ICES policies during data analysis by the designated analyst. All donor registration results will be kept strictly confidential and no one (including the study staff) will know the registration details of any particular patient. In addition, we will not be permitted to report site-level data due to institutional policies. All study-related information will be stored securely at the researcher’s site. For the process evaluation, all participant information will be stored in locked file cabinets in areas with limited access.

### Dissemination policy, authorship eligibility and data sharing plans

We aim to publish our findings regardless of negative or null results. All named authors in the protocol will be offered participation in the final outcomes paper and any subsequent papers. We will not use any professional writing services. Due to institutional policies, we will not be able to grant public access to the participant-level dataset. We may be able to provide a statistical code. We will grant public access to all intervention materials. We will not provide the full transcripts for the process evaluation to protect confidentiality.

## Patient involvement

Our trial is being conducted in line with Canada’s Strategy for Patient-Oriented Research, recognizing that patients and the public provide significant contributions and enhancement to the health research enterprise. We convened a panel of citizen collaborators composed of organ donor recipients, donation advocates, as well as interested citizens without a particular background in organ donation per se. This group contributes their expertise through lived experience, the perspective of which we are targeting with this intervention, as well as their professional experiences. They have contributed, and continue to contribute, to all aspects of the research.

## Discussion

As far as we know, this study will be the first Canadian multi-site cluster randomized controlled trial of an intervention to increase donor registration delivered within family physician offices. As of 2017, organ donor registration rates in Ontario stand at 31%. Increasing donor registration rates in Canada remains an important component of the overall strategy to increase organ donation.

Our trial has several important strengths. First, our study integrates routinely collected administrative healthcare data in a clinical trial design. This population-level approach allows us to enroll and objectively collect behavioral data on *all* patients who visit a family physician clinic. Second, we use a behavior-change, theory-based approach to develop, inform and report our intervention, aiming to provide a novel behavior change-focused strategy to improving registration while simultaneously increasing the transparency and replicability of our intervention. Our intervention targets identified barriers and enablers using behavior-change theory based upon our qualitative work. Our intervention was also designed with substantial input from patients, family physicians, health psychologists and the provincial organ procurement marketing and communications team. Third, as opposed to involving research staff in the delivery, our intervention is entirely delivered by reception staff within family practice settings to ensure consistency with real-world settings.

A potential limitation for this trial is that we are unable to quantify the degree to which our intervention was delivered as intended. We will instruct each reception staff member to provide pamphlets to all patients who have not registered for organ donation upon checking their health card for eligibility. Although we can count the number of pamphlets that were given out, we will not ultimately know how many pamphlets were given to eligible patients (i.e., patients whose health cards did not have the *donor* status). Under the intention-to-treat principle, all patients with visits during the intervention periods will be analyzed as if they were exposed to the intervention.

Another limitation is that we are testing our intervention in an area with a donor registration rate that is relatively higher than the rest of the province, although there still remains substantial room for improvement. Finally, the small number of sites enrolled in our trial limits the generalizability of results, although the offices included in the trial are likely to be representative of a large number of practices across Southwestern Ontario. Prior to embarking on a much larger trial, we deemed it essential to evaluate its effectiveness in a smaller number of settings. Our trial will provide essential information about our intervention and implementation strategy to inform a potential larger (provincial) roll-out. Internal validity may be affected if the participating sites have substantially different outcomes with different trajectories over time; however, the likelihood of differential secular trends over the relatively short duration of the trial is low. Moreover, to improve statistical validity of the mixed-effects regression analyses with a limited number of sites, small-sample, degree-of-freedom corrections will be used.

In conclusion, we developed a theory-informed intervention addressing barriers and enablers to donor registration identified via our previous work. We will test it using a cluster randomized, stepped-wedge design. The results of RegisterNow-1 should help inform future strategies to promote organ donor registration within the family physician setting.

## Additional files

Additional file 1: Table S1. SPIRIT Checklist. (DOC 121 kb)

Additional file 2: Table S2. Intervention Table. (DOCX 29 kb)
