# Supplementary material for: Promoting deceased organ and tissue donation registration in family physician waiting rooms (RegisterNow-1 trial): study protocol for a pragmatic, stepped-wedge, cluster randomized controlled registry
Source: Trials. 2017 Dec 21;18:610. doi: 10.1186/s13063-017-2333-5 (PMC5740738; doi:10.1186/s13063-017-2333-5)
Supplement: Supplementary file 2 — Intervention Table. (DOCX 29 kb) [file 13063_2017_2333_MOESM2_ESM.docx]

**Table S2: Intervention description table Promoting Deceased Organ Donor Registration in Family Physician Offices RegisterNow Trial-1**

This document describes how the results from interviews conducted about barriers and enablers to organ donation registration informed the selection of behaviour change techniques used to target those barriers/enablers and how these are delivered. This table was developed from interviews with 20 individuals, of which 9 reported not yet being registered for organ donation and 11 reported being registered.

| **TDF Domain** | **Who delivers, and how, with what message?** | **Behavior Change Techniques** |
| --- | --- | --- |
| **Knowledge** | | |
| Theme 1: Unclear about registration process*.  Description: Respondents were unaware or did not know how to register for organ donation. | **Reception staff:** Check back of card for donor status. If not a donor or if red/white card, provide leaflet and point to iPad or cellphone for online registration.  **Leaflet:** Include steps on how to register to address procedural knowledge | Social support (practical)  Instruction on how to perform the behaviour  Instruction on how to perform the behaviour |
| *“I haven't had an opportunity to talk with anyone or – knowing how to even do it, so it just passed by.”* |  |  |
| *“I don’t know - the first thing of how to register – I wouldn’t have a clue.”* |  |  |
| *“I think they say sign your driver’s license or something but I don’t even have a driver’s license so forget that.”* |  |  |
| Theme 2: Unclear about eligibility*  Description: Respondents were not clear about their eligibility to register for organ and tissue donation. In Ontario, the only eligibility criteria is that you are at least 16 years of age. | **Leaflet:** Include information on eligibility. | Verbal persuasion about capability |
| *“If there was a cut-off date of how old. . .if there was a certain age that they didn’t want your organs”* |  |  |
| Theme 3: Lack of awareness of where to find donor information*  Description: Respondents were not aware that donor information was available on the back of their photo health card. Many believe it was the back of their driver’s license. | **Reception staff:** check back of health card for donor status  **Leaflet**: visual indication of where to check donor status on health card | Instruction on how to perform the behaviour  Instruction on how to perform the behaviour |
| *Interviewer: “So, if we actually look on the back of your health card is where it will be now”*  *Respondent: “Oh, really?”*  *Interviewer: “…So are you yourself a registered organ donor”?*  *Respondent: “I think I possibly was, but I don’t think I am any more. I don’t remember.”*  *Interviewer: “So it should be on the back of your health card.”*  *Respondent: “Oh, health? I thought it was maybe on the driver’s license”* |  |  |
| **Skills** | | |
| Theme 1: Literacy  Description: Respondents answered that literacy and English skills were necessary to register. | **Leaflet:** Ensure that the content is written for a general audience | n/a |
| Theme 2: Computer skills  Description: Some respondents were not confident about their skills regarding registering online. | **Leaflet:** Provide clear instructions in leaflet for how to register online | Instruction on how to perform the behaviour |
| *“I’m not that gifted on computers. I’m 72. I’m really smart in the old ways.”* |  |  |
| **Social/Professional Role and Identity** | | |
| Theme 1: Volunteerism  Description: Respondents felt that organ donation aligned with their views on volunteering  *“Oh yes, I’m very so much so serving others for the good of them, and in turn it comes back to me”* | **Leaflet**: clarify local community impact | Information about social and environmental consequences |
| Theme 2: Identity  Description: Respondents identified with who themselves were with organ donation  *“I thought it was pretty consistent with like how I like be life and everything, so it just made to do it, because it kind of fit in with my values that way.”* | (not addressed) | n/a |
| **Beliefs About Capabilities** | | |
| Theme 1: Respondents were positive about their ability to register  Description:  *“it’s very easy when you renew your health card. It’s just, I don’t even think I had to sign anything extra. You just say yes or no, and it’s done.”* | **Leaflet:** Emphasize the ease of registration with key steps and short amount of time (also using quotes of others saying it was easy), and that can be done while waiting for appointment | Instruction on how to perform the behaviour  Verbal persuasion about capability  Vicarious consequences |
| Theme 2: Poor health*  Description: Respondents noted that their poor health would not make them an attractive donor  *“Like, actually I laugh about it, why would they want my body? I have destroyed it and it’s got two kinds of cancer.”*  *“As I've gotten older, in my past medical history I have had some malignant tumors removed from my bowel and just assumed because of that I wouldn’t qualify anyway.”*  *“Now, whether my particular parts [laughs] would be of much use, you know, depending how old you are, I think, you know, sometimes they're no good anyway.”* | **Leaflet:** Provide info that anyone regardless of medical history can sign up to be a donor. | Verbal persuasion about capability |
| **Optimism** | | |
| Theme 1: Optimism  Description: Respondents were optimistic that becoming an organ donor would benefit others  *“I have confidence it’s a good organization, best of intentions, in many cases or most cases, or whatever, the organs or tissue do get used, and like I said, if they don’t, well, you know, they were there, and that’s all we can do.”* | (not addressed) | n/a |
| **Beliefs about Consequences** | | |
| Theme 1: Benefits to themselves*  Description: Respondents noted that registering for organ donation had some self-benefit  *“You do it for self satisfaction as well as helping.”*  *“Just self satisfaction I guess.”* | **Leaflet:** Feature stories of donor registrants feeling proud and good about registering | Vicarious consequences  Information about emotional consequences |
| Theme 2: Benefits the recipients*  Description: Respondents noted that organ donor registration could benefit others  *“…giving someone else an opportunity to live or to have a new chance on life, like a better quality of life.”*  *“…it could be helpful to someone else - help someone carry on life.”* | **Leaflet:** Feature stories of recipients benefiting | Information about social and environmental consequences  Salience of consequences  Credible source |
| Theme 3: Benefits others*  Description: Respondents noted that organ donor registration could benefit others such as family members  *“like my family will have the knowledge that I did save somebody and in cases where maybe there’s six different people.”*  *“I felt like anyone really could benefit from it really. Like someone’s family members could benefit from it if someone needed a transplant.”*  *“Because it's not only the recipient, there's his whole family or her whole family.”* | **Leaflet:** Feature quotes of family members potentially benefiting | Information about social and environmental consequences |
| **Intentions** | | |
| Theme 1: People think about it but just haven’t done it  *“But personally I think it's fine, I just never have done it.”* | (addressed via other domains) |  |
| **Goals** | | |
| Theme 1: Religion  Description: Goals aligned with religion  *Interviewer: “How consistent is registering for organ donation with your broader goals and values?*  *Respondent: “Well, it fits right in. We’re fairly religious people, and, you know, certainly believe in helping fellow mankind.”* | **Leaflet**: remind of goal consistency with most religions | Information about others’ approval |
| **Memory, Attention and Decision Processes** | | |
| Theme 1: Organ donation is often a passive thought*  Description: Organ donation is in the back of people’s minds  *“It's never approached me in the way that you have, more direct. It's one of the things you keep in the back of your mind that it's a good thing to do.”*  *“And, you know, through my 30s and 40s and 50s I really just didn’t even think about it, you know, which is a shame”*  *“I never got around to it. I've thought about it but I just never have done it.”* | **Reception staff:** Provide leaflet and suggest use of iPad or their cellphone to register if they want to  **Leaflet:** Acknowledge most people get busy and forget, prompt to take 2 minutes to register while they wait for their appointment.  **iPad:** (acts as a prompt/cue in itself) | Prompts/Cues  Social Comparison  Prompts/Cues  Prompts/Cues |
| **Environmental Context and Resources** | | |
| Theme 1: Medical Setting  Description: Working in a medical setting made respondents think about donation more | **iPad:** add an iPad to act as a resource in a new context to enable the behaviour | Adding objects to the environment |
| *“I think because I worked in a trauma unit and I realize the benefits of it”* |  |  |
| **Social Influences** | | |
| Theme 1: Organ donation is mostly personal decision  *“I think it's a personal choice. If you feel like you should do it then you should do it.”* | **Reception staff and leaflet:** Promote that organ donation is a personal choice | Information about others’ approval |
| Theme 2: Knowing other donors*  Description: Knowing a donor or being part of the experience or not  *“I know with even my mom having the organ – the eye donation done they sent a beautiful certificate and …they send a beautiful certificate to the family and they send a beautiful like it’s like a little medallion and stuff like that and my dad still has it to this very day sitting on his thing and that meant a lot to him to say that, you know, she did something.”*  *“I don't think I do. That’s not a conversation that you have really, you know.”*  *“I can't think even of a family member who may have registered that I'm aware of.”* | **Leaflet:** Feature members of local community who became a donor and who benefitted from donation, and family physicians in the office | Credible source  Salience of consequences  Information about social and environmental consequences  Social comparison |
| **Emotion** | | |
| Theme 1: Positive emotion* | **Leaflet:** provide quote from someone locally about positive emotion felt | Vicarious consequences  Information about emotional consequences |
| *“A little self gratification I guess. It's going to a good purpose.”*  *“There's a lot of self satisfaction of providing something that you no longer need that could help people down the road.”*  *Interviewer: “So can you pick an emotion, like?”*  *Respondent: “Probably would be uplifting.”* |  |  |
| Theme 2: Negative emotions  *“It is a daunting thought in some ways.”* | (not addressed in this intervention) |  |
| Theme 3: No Emotion*  Description: Some respondents reporting feeling no emotion  *“Well, not really any. This might sound hardened, but not really any emotion sort of type thing..”*  *Interviewer: “So how does the overall concept of registering for organ donation make you feel?”*  *Respondent: “Fine. No issues”* | (not addressed in this intervention) |  |
| **Behavioural Regulation** | | |
| Theme 1: Needs to be simple and automatic  *“So it was kind of more of a laziness thing. I think the simpler you can make the registration, the more easy and automatic that is, more people will do it.”* | **iPad**: (providing iPad in the setting simplifies and automates many steps) | Adding objects to the environment |
| **Reinforcement** | | |
| Theme 1: Incentives*  Description: Respondents reacted negatively to monetary or financial incentives  *Interviewer: “…would you desire or want or need any incentive to donate or to register?”*  *Respondent: “No.”*  *Interviewer: “No. Do you think others need it?”*  *Respondent: “I would hope not. Do you mean, like, incentive like, almost like an exchange, you give them something or?”*  *Interviewer: “Something or a reward or something, yeah”.*  *Respondent: “Oh, I would hope not.”*  *Interviewer: “Yeah. So, some people join here for 20 bucks, right or whatever.”*  *Respondent: “No, I would hope you wouldn't do it for that.”* | (informed decision not to offer financial incentives) |  |
| **Intervention Suggestions by participants** | | |
| Theme 1: Physician Office*  Description: Respondents thought physician offices were appropriate  *“This is a good spot. They're very loyal… they're very confidential people.”* | (informed the setting selection) |  |
| Theme 2: Discussion*  Description: Respondents thought that having a discussion with someone was important  *“I would rather - I want to talk to the person..”*  *“Talk to them.”* | **Leaflet**: letter from practice team encouraging patients to discuss their decision with their health team and/or their family | Credible source |
| Asterisk (*) indicates that more than one respondent indicated this. | | |
